# Evaluating type and amount of dietary protein in relation to metabolic syndrome among Iranian adults: cross-sectional analysis of Fasa Persian cohort study

**DOI:** 10.1186/s13098-022-00813-0

**Published:** 2022-03-18

**Authors:** Ali Jamshidi, Mojtaba Farjam, Maryam Ekramzadeh, Reza Homayounfar

**Affiliations:** 1 Student Research Committee, Shiraz University of Medical Sciences, Shiraz, Iran; 2 Noncommunicable Diseases Research Center, Fasa University of Medical Sciences, Fasa, Iran; 3 Nutrition Research Center, Department of Clinical Nutrition, School of Nutrition and Food Sciences, Shiraz University of Medical Sciences, Shiraz, Iran; 4 National Nutrition and Food Technology Research Institute, Faculty of Nutrition Sciences and Food Technology, Shahid Beheshti University of Medical Sciences, Tehran, Iran

**Keywords:** Metabolic syndrome, Dietary protein, Animal protein, Plant protein, Abdominal obesity, Hypertension, Dyslipidemia, Glucose intolerance

## Abstract

**Background and aim:**

Metabolic syndrome is characterized by several conditions including abdominal obesity, dyslipidemia, impaired glucose levels, and hypertension; which all are directly associated with an increased risk of cardiovascular disease and type 2 diabetes mellitus. This study aimed to evaluate the association of the amount and source of dietary protein with the risk of metabolic syndrome and its components in adult men and women.

**Material and method:**

This study was performed using Persian cohort data, Fasa branch, which included 3660 men and 5262 women. Individuals were divided into five groups for total dietary proteins, plant proteins, and animal proteins using the quintiles, and the odds of incidence for metabolic syndrome and each component was evaluated.

**Results:**

A higher intake of total protein was associated with lower odds of having metabolic syndrome (OR: 0.24 95% CI 0.18–0.33, P-trend < 0.001 in men, OR: 0.42 95% CI 0.34–0.51, P-trend < 0.001 in women) and all of its components. men (OR: 0.35 95% CI 0.25–0.48, P-trend < 0.001) and women (OR: 0.41 95% CI 0.33–0.52, P-trend < 0.001) in the highest quintile of plant protein intake had a reduced prevalence of metabolic syndrome and its components. An increased animal protein intake was associated with a lower prevalence of metabolic syndrome (P-trend < 0.001), a declined risk of having elevated triglycerides (P-trend = 0.016) in men, and a reduced risk of having abdominal obesity in men (P-trend < 0.001) and women (P-trend < 0.001).

**Conclusion:**

A higher consumption of total protein and plant protein was associated with a lower prevalence of metabolic syndrome and its components. Increased consumption of animal protein seemed to be related to a lower prevalence abdominal obesity. Also, only in men, animal protein was associated with decreased risk of having metabolic syndrome.

## Introduction

Chronic diseases such as hypertension, diabetes, and dyslipidemia, are among the most prevalent and costly health issues and they are considered as the leading cause of morbidity and mortality in the world. Chronic diseases can be prevented by controlling or treating risk factors such as overweight, and obesity, as well as unhealthy eating habits, physical inactivity, and psychosocial conditions [1]. Metabolic syndrome (MetS), formerly known as Syndrome X, is a complex disorder that has become a worldwide epidemic with high socioeconomic costs. The syndrome is related to a range of diseases, including abdominal obesity, dyslipidemia, insulin resistance, glucose intolerance, hypertension, and endothelial dysfunction, which are directly associated with an increased risk of coronary heart disease, other forms of cardiovascular disease, and type 2 diabetes mellitus [2, 3].

In 2009, a consensus was attained for the diagnostic criteria of metabolic syndrome by six organizations including the International Diabetes Federation (IDF), the American Heart Association, the National Heart, Liver, and Blood Institute, the World Heart Federation, the International Atherosclerosis Society, and the International Association for the Study of Obesity. According to the consensus, MetS can be defined if any three or more of the following criteria are diagnosed in a patient: (i) Elevated blood pressure: systolic ≥ 130 and/or diastolic ≥ 85 mmHg; (ii) Increased fasting Blood Glucose ≥ 100 mg/dl; (iii) Elevated blood triglycerides ≥ 150 mg/dl; (iv) Decreased high-density lipoprotein cholesterol (HDL-c) ≤ 40 mg/dl for men, or ≤ 50 mg/dl for women; and (v) Elevated waist circumference based on population- and country-specific cut-off points [4, 5].

The global prevalence of MetS is between 14 and 32% among men and women, which increases with aging. For instance, the prevalence of MetS is 28.8% in Turkey, 25.9% in Denmark and 25% in the United States. According to available data, the prevalence of MetS in Iranian adults over the age of 20 is 33.7%, which is more prevalent among women than men (42% vs. 24%) [6].

The prevalence of non-communicable diseases is increasing in the developing countries as a result of nutritional, demographic, epidemiological, and socioeconomic transitions. Urbanization is the major driver for nutrition transition. Immigration from rural to urban expose migrants to urbanized diets and lifestyle [7]. Evolutionarily, our ancestors had to survive periods of famine; so, as the result of imposed pressure, the genotype of overeating, lower energy expenditure, and physical inactivity increased. Moreover, we are living in obesogenic environments now which affect our behavior and lifestyle choices and it has increased the prevalence of obesity over the last 50 years. Some of these habit changes (which is known as Westernization of lifestyles) can be addressed as decreased eating of homemade foods, higher usage of air conditioning which can lower energy expenditure to sustain body temperature, decreased physical activity, and a growing habit of eating snacks [8]. The global prevalence of Mets could be associated with increased consumption of high-calorie low-fiber foods, Sedentary conduct and decreased physical activity in societies [9], in addition to excessive consumption of alcohol, saturated fat, ultra-processed foods and added sugar [10–12]. Psychosocial stress can also play a role in most components of metabolic syndrome, as the metabolic syndrome indicators seem to be more prevalent in deprived communities [11]. Food industries also have major roles in creating an environment where the avoidance of high-calorie foods is inevitable by producing and promoting inexpensive sweetened beverages and ultra-processed foods high in salt, sugar, and saturated fat, which only evoke a transient feeling of satiety [13].

Obesity itself can cause insulin resistance, hypertension, and dyslipidemia. Several studies have shown an association between obesity, especially abdominal obesity, with impaired insulin sensitivity and type 2 diabetes [14]. On the other hand, previous studies have shown hyperinsulinemia has a causal role in adipose tissue growth, as pharmacological reduction of insulin secretion can decrease body weight in people with obesity. [15]. Hyperinsulinemia also is considered as a driving factor in obesity, insulin resistance and diabetes. It is demonstrated that preventing hyperinsulinemia can prevent obesity in euglycemic status [16]. Moreover, there is a strong positive correlation between body mass index and hypertension. So that, up to 30% of the occurrence of hypertension can be associated with obesity. Obesity is also a factor in increasing blood triglycerides and lowering HDL-C levels [14].

Unhealthy dietary habits, sedentary lifestyle, and low socio-economic status among people lead to a rise in the prevalence of metabolic syndrome in developed countries. Thus, finding practical solutions to identify, treat and control metabolic syndrome and its associated comorbidities has become a crucial concern [17]. Several studies have demonstrated the role of lifestyle changes such as increasing physical activity, following a healthy eating pattern, and losing weight in reducing the risk of metabolic syndrome and its components. Various factors, such as a high-calorie high-saturated fatty acid diet and a high-carbohydrate diet, can be linked to obesity, insulin resistance, and metabolic syndrome [18, 19]. Adherence to a diet reach in fish, fruit and vegetables results in a healthier metabolic profile and it reduces the risk of MetS. Consumption of whole-grain cereals, rather than processed cereals, also seems to be protective against metabolic syndrome [20, 21]. Regarding the role of protein in the development or prevention of metabolic syndrome, the results are inconsistent, and the proper amount or source of protein for improving insulin resistance and metabolic syndrome is unclear. Protein intake can be associated with increased satiety, reduced lean tissue loss, decreased glycemic response, decreased gastric emptying rate, and increased insulin secretion, which all could be effective in controlling metabolic syndrome [22–24].

The various effects of protein on metabolic syndrome may be due to the differences in protein quantity and quality. Dietary protein is divided into two categories based on its source: animal-derived protein and plant-based protein, which differ in structure, function, amino acid composition, and digestibility (bioavailability) [25]. Plant proteins account for a lower source of methionine and branched-chain amino acids (BCAA) in comparison with animal proteins. It was shown that a methionine-restricted diet could prevent the development of the metabolic syndrome and insulin resistance through the protective fibroblast growth factor 21 pathway in animal studies. Furthermore, BCAA could induce insulin resistance by inhibiting mitochondrial ATP production. Therefore, It seems that consumption of plant protein has some beneficial effects on metabolic responses [26].

A better understanding of the role of protein and its sources in obesity and metabolic syndrome leads to more effective prevention and control of this syndrome in individuals. This study aimed to evaluate the amount of total, animal, and vegetable dietary protein intake and assess their relationship with anthropometric indices and metabolic syndrome variables (abdominal obesity, hypertension, impaired glucose level, increased serum triglyceride, and decreased high-density lipoprotein).

## Material and methods

### Study subjects

This study was cross-sectional in design, which performed using the data of the Fasa Cohort Study as a branch of the Prospective Epidemiological Research Study in Iran (PERSIAN) cohort [27], designed to examine factors predicting chronic non-communicable diseases. Detailed protocol of the Fasa Cohort Study is reported previously [28]. It was performed during November 2014 and June 2019. In summary, among a total of 41,000 persons resident in rural regions of Sheshdeh, a district from Fasa County, Fars, Iran, 11,097 individuals aged 35 to 70 years were included. Participants were recruited from urban settings and entered into the study by multistage cluster sampling. Subjects were included if they were aged 35–70 years, lived in Sheshdeh district ≥ 9 months each year, and were categorized as Iranian nationality. Also, exclusion criteria were set as people who were reluctant to participate, people with physical or mental disabilities, and people with communication disorders who were not able to answer the study questions. This study was performed using the database which included three sections: general, medical, and nutritional interviews. In summary, in the public interview section, personal information, general information, socio-economic status, employment status, health behaviors, and anthropometric assessments were collected. The medical interview included gathering information such as medical history and medication use, physical activity, and smoking. In the nutritional section, dietary habits and amount of food consumption were collected using a semi-quantitative questionnaire of 125-item food frequency, which was adapted to Iranian foods. In this method, individuals were asked to report the frequency of consumption of the requested foods in the past year based on serving sizes daily, weekly, or annually. All data collection was done by trained personnel. All individuals participating in the study signed written consent. The Ethics Committee of Shiraz University of medical sciences, Shiraz, Iran approved the protocol of the study (IR.SUMS.REC.1399.1029).

Among the 11,097 available data, people with the age of 35 to 70 years, with complete database (anthropometric information, personal and general information, nutrition information, drug and disease information, laboratory data) and daily energy intake between 500 and 5000 cal were selected as criteria for inclusion in the study. Pregnant women and individuals with disabilities hindering them to stand upright for anthropometric measurements were excluded. Finally, a total of 8922 individuals, including 3660 men and 5262 women were included in this study.

### Definition of metabolic syndrome

Modified National Cholesterol Education Programs Adult Treatment Panel III (NCEP ATP III) [29] and IDF [4] guidelines were used for the diagnosis of metabolic syndrome as the existence of at least three of the five criteria in an individual. Criteria were determined as abdominal obesity, increased blood triglycerides (≥ 150 mg/dl or take medication), decreased high-density lipoprotein (≤ 40 mg/dl in men and ≤ 50 mg/dl in women), increased blood pressure (≥ 130 mmHg for systolic blood pressure and/or ≥ 85 mmHg for diastolic blood pressure or taking antihypertensive drugs); And impaired fasting glucose (≥ 100 mg/dl or drug therapy). According to the guidelines, the criteria for abdominal obesity should be adjusted and personalized based on population and country-specific data. The recommended waist circumference cut-point is ≥ 80 cm for Asian women and either ≥ 90 or ≥ 94 cm for Asian men depending on the region and the availability of related data [5]. Previous studies on Iranians have reported inconsistent cut-points for Iranian men, such as ≥ 87.5 cm [30] and ≥ 91 cm [31]. Therefore, we considered the nearest IDF cut-point [4, 5] (≥ 80 cm for women and ≥ 90 cm for men) to these findings, as the most suitable cut-point for this study.

### Nutritional assessment

The nutritional evaluation was performed by using a the Willett format and validated semi-quantitative questionnaire of 125-item food frequencies (FFQ) [32] adapted to Iranian food. Individuals were requested face to face by an expert nutritionist to report their frequency of consumption of the given serving of each food item during the past year, on a daily, weekly, monthly, or yearly basis. The standard portion size was designed for each item by using United States Department of Agriculture serving sizes (such as bread, one slice; diary, one cup). The mean daily energy and nutrient contents of foods were calculated using the Nutritionist IV software (version 7.0) by multiplying the consumption frequency of each food item by its nutrient content and then summing across all foods. Protein intake was divided into two sources of animal protein and plant protein. The source of animal protein in the diet was considered as red meat, poultry, fish and seafood, processed meats, bird eggs such as eggs, milk, and dairy products. Plant protein sources included cereals, edible grains, legumes (namely peas, beans, soybeans, and lentils), roots (Such as potatoes and carrots), oilseeds, mushrooms, fruits and vegetables, and refined cereal products such as bread, pasta, and noodles.

### Laboratory evaluations

After 10 to 14 h of fasting, a 25 ml blood sample was taken and stored in coded containers. The samples were then transported to the laboratory by a cool box for further testing. Laboratory tests included complete blood count, fasting blood sugar, total serum cholesterol, triglyceride, high-density lipoprotein, and low-density lipoprotein. All steps were performed by professional personnel and advanced sampling tools. TC and HDL were measured by using the Mindray BS380 autoanalyzer (Mindray Medical International, Shenzhen, China) as the biochemical tests. Laboratory testing of FBS was obtained using glucose oxidize test. We used the Friedewald formula to calculate LDL [33]. Whenever TG was greater than 400 mg/dl, the LDL-C was measured by the direct method instead of LDL calculation [34] using Mindray BS380 autoanalyzer.

### Anthropometric and clinical assessments

Anthropometric and clinical data were collected by trained and professional specialists. Anthropometric measurements were performed according to the measurement protocol of the US National Institutes of Health. Weight and height were measured using a digital scale and body mass index (BMI) was calculated by dividing the weight (kg) by height squared (m^2^). Waist circumference and hip circumference were measured with the use of a constant tension tape with a precision of 0.1 cm at the level of the umbilicus and the widest point over the buttocks, respectively. Blood pressure was measured twice with an interval of 15 min in a sitting position and after at least 5 min of rest using a sphygmomanometer (Sphygmomanometer, mercury, ALPK1, Japan). Moreover, a bioelectric impedance analysis (BIA) system (Tanita BC-418, Tanita Corp, Japan) was added to the study in the second phase as an additional measurement. The anthropometric data of all the 4177 participants in the second phase was measured by BIA, including 1758 men and 2419 women.

### Other covariates

Socio-demographic data such as age, gender, wealth index, level of education, health-related behaviors such as smoking, drinking alcohol, and physical activity were collected through an interview. Physical activity was calculated based on the metabolic equivalent of tasks calculated in 24 h through the International Physical Activity Questionnaire (IPAQ).

### Data analysis

Individuals were separated into two groups according to gender, and then each group was divided into quintiles, based on the amount of protein intake per body weight for each of the total protein, animal protein, and plant protein intakes.

Categorical variables of general characteristics were described as frequencies and proportions and compared by sex using the chi-square test. Continuous variables were expressed as means and their standard errors and compared by sex using Independent Sample t-test.

ANCOVA test was performed to determine the relationship between total protein intake and anthropometric indices, blood tests, and dietary intakes in both men and women, and the results were reported as means and standard error. In the basic model, the test was performed by considering the variables of age, physical activity, and wealth index as adjusting factors for all variables. In adjusted models, in addition to basic model modifiers, potential covariates based on the variable were added.

Odds ratio (OR) and 95% confidence interval (CI) were calculated using logistic regression test to determine the association between total protein, animal protein, and plant protein intake with metabolic syndrome and its components in both men and women. The first time, total protein was considered as an independent variable and in the second test, animal protein and plant protein were considered as the main variable simultaneously. For all components, in addition to age, physical activity, and wealth index, depending on the nature of each component, the potential adjustments were applied. Moreover, the P-trend was obtained by considering the median intake of total, animal and plant protein as a continuous variable in groups.

As a sub-analysis, for individuals whose anthropometric data were measured by BIA, the relationship between total protein intake and fat mass, lean mass, and fat percentage was determined by ANCOVA, reported as mean and standard error. Physical activity, age and wealth index were considered as confounding factors.

All tests were performed using SPSS software version 26 and P-value less than 0.05 was considered as a significant level.

## Results

### General characteristics of participants

The general characteristics of the subjects according to gender are shown in Table 1. Women accounted for 59% and men 41% of the study population and were age-similar (P = 0.264). Mean of height and weight were higher in men than women, while body mass index, waist circumference, hip circumference, and waist to hip circumference ratio were higher in women (P < 0.001). The mean of waist circumference in women and men was 96.08 and 89.23 cm, respectively, which was above the criteria of metabolic syndrome in women.

**Table 1:** Characteristics of participants according to gender (SE)

	Men	Women	Ρ-value
N (%)	3660 (41%)	5262 (59%)	
Age (year)	48.95 (0.159)	48.72 (0.132)	0.264
Height (cm)	168.75 (0.107)	155.69 (0.078)	< 0.001
Weight (cm)	68.669 (0.224)	65.114 (0.173)	< 0.001
BMI (kg/m^2^)	24.07 (0.072)	26.83 (0.066)	< 0.001
Waist circumference (cm)	89.23 (0.181)	96.08 (0.159)	< 0.001
Hip circumference (cm)	97.25 (0.124)	101.19 (0.129)	< 0.001
Waist to hip ratio	0.91 (0.001)	0.94 (0.000)	< 0.001
Systolic blood pressure (mmHg)	110.39 (0.292)	111.93 (0.265)	< 0.001
Diastolic blood pressure (mmHg)	74.14 (0.196)	74.78 (0.167)	0.013
Blood glucose (mg/dl)	90.37 (0.387)	94.71 (0.459)	< 0.001
Triglycerides (mg/dl)	135.89 (1.481)	127.47 (0.971)	< 0.001
Cholesterol (mg/dl)	178.97 (0.626)	190.19 (0.541)	< 0.001
HDL-cholesterol (mg/dl)	46.96 (0.231)	54.03 (0.222)	< 0.001
LDL-cholesterol (mg/dl)	104.83 (0.519)	110.66 (0.456)	< 0.001
Smoking (n-%)			
Overall	1942 (51.3%)	263 (5.0%)	< 0.001
Current-smoker	1423 (38.9%)	122 (2.3%)	< 0.001

Nutritional intake/day (SE)			
Energy (kcal/day)	3389.30 (13.939)	2855.45 (11.730)	< 0.001
Carbohydrate (%e)	67.91% (0.097)	67.91% (0.080)	0.977
Fat (%e)	20.04% (0.098)	20.19% (0.083)	0.259
Total protein (%e)	12.03% (0.027)	11.89% (0.022)	< 0.001
Animal protein (%e)	3.51% (0.026)	3.12% (0.021)	< 0.001
Plant protein (%e)	8.52% (0.024)	8.76% (0.020)	< 0.001
Energy (gr/kg_bw_)	50.42 (0.253)	44.67 (0.216)	< 0.001
Carbohydrate (g/kg_bw_)	8.66 (0.045)	7.70 (0.038)	< 0.001
Fat (gr/kg_bw_)	1.13 (0.008)	1.01 (0.006)	< 0.001
Total protein (gr/kg_bw_)	1.52 (0.008)	1.34 (0.006)	< 0.001
Animal protein (gr/kg_bw_)	0.44 (0.003)	0.34 (0.002)	< 0.001
Plant protein (gr/kg_bw_)	1.08 (0.006)	1.00 (0.005)	< 0.001
Carbohydrate (gr/day)	575.95 (2.539)	485.75 (2.124)	< 0.001
Fat (gr/day)	75.29 (0.482)	63.66 (0.361)	< 0.001
Total protein (gr/day)	101.96 (0.479)	84.88 (0.382)	< 0.001
Animal protein (gr/day)	29.44 (0.250)	21.93 (0.165)	< 0.001
Plant protein (gr/day)	72.51 (0.382)	62.94 (0.318)	< 0.001
Sodium (mg/day)	4019.74 (22.450)	3745.49 (20.038)	< 0.001
Potassium (mg/day)	3700.23 (26.206)	3297.05 (20.008)	< 0.001
Saturated fatty acids (gr/day)	28.35 (0.238)	24.90 (0.190)	< 0.001
MUFA (gr/day)	22.86 (0.174)	18.52 (0.119)	< 0.001
PUFA (gr/day)	10.51 (0.081)	8.89 (0.060)	< 0.001

Data are reported as mean and standard error for continuous variables, and as proportion and percentage for categorical variables

P-values were derived from the Chi-Squared test for categorical variables and Independent sample t-test for continuous variables represents differences between men and women

The significance level was considered P < 0.05

*BMI* body mass index, *HDL-c* high-density lipoprotein, *LDL-c* low-density lipoprotein, *MUFA* monounsaturated fatty acids, *PUFA* polyunsaturated fatty acids

Women were more likely to have higher systolic blood pressure (P < 0.001), diastolic blood pressure (P = 0.013), fasting glucose level (P < 0.001), total cholesterol (P < 0.001), LDL-c (P < 0.001), and HDL-c (P < 0.001) than men. In contrast, the serum levels of triglyceride were higher in men (P < 0.001), and they tended to smoke more than women (P < 0.001).

Men consumed more energy (kcal/day), carbohydrate (gr/kg BW), protein including both plant and animal protein (gr/kg BW), fat and fatty acids(gr/kg BW), sodium and potassium (mg/day) (P < 0.001) compared to women. There was no difference between men and women regarding the percentage of dietary energy intake from carbohydrates (men: 67.91% and women: 67.91%, P = 0.977) and fat (men: 20.04% and women: 20.19%, P = 0.259). But the percentage of energy intake from total protein, animal protein, and plant protein was higher in men (P < 0.001).

### Presence of metabolic syndrome components according to genders

Table 2 shows the distribution of metabolic syndrome and its components in the subjects by gender. The percentage of cases diagnosed with metabolic syndrome was higher in women than men (40.4% vs. 25.1%, respectively, P < 0.001), which means that at least three indicators of metabolic syndrome were observed in them. Among the components of metabolic syndrome, abdominal obesity was the most common in both genders. According to the criteria of metabolic syndrome, 84.1% of women and 48.3% of men had abdominal obesity (P < 0.001). Elevated triglyceride levels was the only indicator that was not statistically different between men (33%) and women (32.5%) (P = 0.614).

**Table 2:** Prevalence of metabolic syndrome and its components among the subjects by gender

Metabolic criteria (n-%)	Men	Women	P-value
Hypertension	1028 (28.9%)	2036 (39.7%)	< 0.001
Impaired blood glucose	557 (15.2%)	1284 (24.4%)	< 0.001
Elevated waist circumference	1766 (48.3%)	4457 (84.1%)	< 0.001
Increased triglycerides	1188 (32.5%)	1735 (33%)	0.614
Decreased HDL-c	1257 (34.3%)	2544 (48.3%)	< 0.001
Has metabolic syndrome	918 (25.1%)	2133 (40.5%)	< 0.001
Number of criteria			
0	904 (24.7%)	320 (6.1%)	
1	1004 (27.4%)	1153 (21.9%)	
2	834 (22.8%)	1656 (31.5%)	
3	609 (16.6%)	1204 (22.9%)	
4	248 (6.8%)	696 (13.2%)	
5	61 (1.7%)	233 (4.4%)	

Values are reported as proportion and percentage

P-values were derived from the Chi-Squared test to represent differences between men and women

The significance level was considered P < 0.05

*HDL-c* high-density lipoprotein

### Baseline characteristics according to the quintiles of total dietary protein intake per weight

Baseline characteristics according to the quintiles of total dietary protein intake per weight were compared in both genders in Table 3. There was a negative association between total protein intake and body weight, body mass index, waist circumference, hip circumference, and waist-to-hip ratio in men and women (P < 0.001). Total protein intake was inversely related to mean systolic (P < 0.001) and diastolic (P < 0.001) blood pressure levels in men but not any significant correlation was found in women in systolic (P = 0.259) and diastolic (P = 0.199) blood pressure after adding adjustments. Fasting blood sugar levels was not significantly associated with the amount of protein intake in men (P = 0.157) and women (P = 0.329). Total cholesterol, LDL-c, and serum triglycerides were negatively related, and HDL-c was positively associated with total protein intake in men (For all P < 0.001). In women increased protein intake was associated with lower serum levels of triglycerides (P = 0.005) and LDL-c (P < 0.001), and higher HDL-c level (P < 0.001). No significant association was found between serum total cholesterol and total protein intake in women.

**Table 3:** Characteristics according to quintiles of total dietary protein intake per kg body weight (mean-SE)

Men						
Total protein						
Characteristics of participants (SE)	Q1	Q2	Q3	Q4	Q5	P-value
N = 3659	731	732	732	732	732	
Total protein (g/kg bw/day)	0.906 (0.00)	1.234 (0.00)	1.485 (0.00)	1.750 (0.00)	2.268 (0.00)	< 0.001
Age (year)	50.00 (0.36)	48.85 (0.35)	49.15 (0.35)	48.25 (0.35)	48.50 (0.35)	0.006
Weight (kg)	75.98 (0.44)	72.51 (0.44)	68.93 (0.44)	65.80 (0.44)	60.10 (0.44)	< 0.001
BMI (kg/m^2^)	26.36 (0.14)	25.18 (0.14)	24.10 (0.14)	23.31 (0.14)	21.42 (0.14)	< 0.001
Waist circumference (cm)	94.79 (0.36)	92.03 (0.36)	89.30 (0.36)	87.14 (0.36)	82.89 (0.36)	< 0.001
Hip circumference (cm)	100.92 (0.25)	99.24 (0.25)	97.45 (0.25)	95.82 (0.25)	92.80 (0.25)	< 0.001
Waist to hip ratio	0.937 (0.00)	0.926 (0.00)	0.914 (0.00)	0.908 (0.00)	0.892 (0.00)	< 0.001
Systolic blood pressure (mmHg)	113.05 (0.61)	111.10 (0.61)	110.31 (0.61)	110.04 (0.61)	107.45 (0.61)	< 0.001
Adjusted*	112.20 (0.59)	110.65 (0.58)	110.24 (0.58)	110.40 (0.58)	108.44 (0.59)	0.001
Diastolic blood pressure (mmHg)	75.99 (0.43)	74.85 (0.43)	74.43 (0.42)	73.63 (0.43)	71.77 (0.43)	< 0.001
Adjusted*	75.40 (0.42)	74.54 (0.41)	74.39 (0.41)	73.84 (0.41)	72.50 (0.42)	< 0.001
Blood glucose (mg/dl)	91.67 (0.85)	92.78 (0.84)	89.57 (0.84)	89.93 (0.84)	87.88 (0.85)	0.001
Adjusted^†^	90.61 (0.75)	91.87 (0.75)	89.76 (0.74)	90.28 (0.74)	89.30 (0.76)	0.158
Triglycerides (mg/dl)	146.53 (3.28)	146.28 (3.27)	133.21 (3.26)	136.10 (3.26)	117.31 (3.28)	< 0.001
Adjusted^‡^	146.21 (3.27)	145.30 (3.25)	134.02 (3.24)	136.14 (3.24)	117.76 (3.28)	< 0.001
Total cholesterol (mg/dl)	183.38 (1.40)	181.53 (1.39)	176.12 (1.39)	180.53 (1.39)	173.26 (1.40)	< 0.001
Adjusted‡	183.46 (1.38)	181.24 (1.37)	176.14 (1.37)	180.64 (1.37)	173.35 (1.38)	< 0.001
HDL-cholesterol (mg/dl)	45.94 (0.50)	45.13 (0.50)	46.65 (0.50)	48.11 (0.50)	48.96 (0.51)	< 0.001
Adjusted^‡^	45.74 (0.50)	45.13 (0.50)	46.61 (0.50)	48.16 (0.50)	49.16 (0.51)	< 0.001
LDL-cholesterol (mg/dl)	108.13 (1.16)	107.14 (1.15)	102.82 (1.15)	105.19 (1.15)	100.83 (1.16)	< 0.001
Adjusted^‡^	108.48 (1.14)	107.05 (1.13)	102.72 (1.13)	105.25 (1.13)	100.63 (1.14)	< 0.001
Nutritional intake						
Energy (kcal/day)	2454.45 (21.96)	3085.88 (21.89)	3452.38 (21.85)	3750.97 (21.86)	4202.14 (22.00)	< 0.001
Carbohydrate %E	67.99 (0.21)	68.22 (0.21)	67.91 (0.21)	67.69 (0.21)	67.74 (0.21)	0.428
Fat %E	20.66 (0.21)	20.09 (0.21)	20.12 (0.21)	19.95 (0.21)	19.41 (0.21)	0.002
Total protein %E	11.33 (0.05)	11.67 (0.05)	11.96 (0.05)	12.35 (0.05)	12.84 (0.05)	< 0.001
Animal protein %E	3.33 (0.05)	3.32 (0.05)	3.43 (0.05)	3.62 (0.05)	3.83 (0.05)	< 0.001
Plant protein %E	7.99 (0.05)	8.35 (0.05)	8.53 (0.05)	8.73 (0.05)	9.01 (0.05)	< 0.001

Women						
Total protein						
Characteristics of participants (SE)	Q1	Q2	Q3	Q4	Q5	P-value
N = 5260	1052	1053	1052	1051	1052	
Total protein (g/kg bw/day)	0.73 (0.00)	1.04 (0.00)	1.27 (0.00)	1.56 (0.00)	2.11 (0.00)	< 0.001
Age (year)	50.11 (0.29)	48.86 (0.29)	48.44 (0.29)	47.74 (0.29)	48.46 (0.29)	< 0.001
Weight (kg)	72.19 (0.34)	68.39 (0.34)	65.34 (0.34)	62.40 (0.34)	57.24 (0.34)	< 0.001
BMI (kg/m^2^)	29.15 (0.13)	28.01 (0.13)	26.93 (0.13)	25.87 (0.13)	23.82 (0.13)	< 0.001
Waist circumference (cm)	102.05 (0.32)	98.43 (0.32)	96.26 (0.32)	94.09 (0.32)	89.56 (0.32)	< 0.001
Hip circumference (cm)	106.41 (0.26)	103.17 (0.26)	101.37 (0.26)	99.16 (0.26)	95.86 (0.26)	< 0.001
Waist to hip ratio	0.959 (0.00)	0.954 (0.00)	0.949 (0.00)	0.948 (0.00)	0.934 (0.00)	< 0.001
Systolic blood pressure (mmHg)	112.66 (0.55)	112.92 (0.55)	112.50 (0.54)	111.32 (0.55)	110.25 (0.55)	0.003
Adjusted*	111.79 (0.53)	112.75 (0.52)	112.32 (0.51)	111.57 (0.52)	111.23 (0.53)	0.259
Diastolic blood pressure (mmHg)	75.23 (0.36)	75.42 (0.36)	75.16 (0.36)	74.48 (0.36)	73.59 (0.36)	0.002
Adjusted*	74.69 (0.36)	75.32 (0.35)	75.06 (0.35)	74.64 (0.35)	74.16 (0.36)	0.199
Blood glucose (mg/dl)	95.77 (1.00)	94.46 (1.00)	95.78 (1.00)	94.66 (1.00)	92.87 (1.00)	0.229
Adjusted^†^	94.14 (0.87)	94.07 (0.87)	95.94 (0.86)	95.49 (0.87)	93.91 (0.87)	0.329
Triglycerides (mg/dl)	129.93 (2.15)	130.52 (2.14)	128.33 (2.14)	127.20 (2.14)	121.37 (2.15)	0.022
Adjusted^‡^	130.31 (2.15)	131.10 (2.12)	128.39 (2.11)	127.03 (2.12)	120.52 (2.15)	0.005
Total cholesterol (mg/dl)	191.01 (1.19)	189.96 (1.19)	190.78 (1.19)	191.03 (1.19)	188.06 (1.19)	0.354
Adjusted^‡^	191.74 (1.18)	190.43 (1.16)	190.49 (1.16)	190.83 (1.16)	187.35 (1.18)	0.102
HDL-cholesterol (mg/dl)	52.07 (0.49)	52.66 (0.49)	53.99 (0.49)	54.95 (0.49)	56.43 (0.49)	< 0.001
Adjusted^‡^	51.77 (0.49)	52.54 (0.49)	53.97 (0.48)	55.05 (0.49)	56.77 (0.49)	< 0.001
LDL-cholesterol (mg/dl)	112.95 (1.01)	111.19 (1.01)	111.11 (1.01)	110.64 (1.01)	107.35 (1.01)	0.003
Adjusted^‡^	113.90 (0.99)	111.67 (0.98)	110.83 (0.98)	110.37 (0.98)	106.47 (1.00)	< 0.001
Nutritional intake						
Energy (kcal/day)	1939.96 (17.09)	2492.84 (17.06)	2827 (17.03)	3229.99 (17.06)	3787.74 (17.12)	< 0.001
Carbohydrate %E	67.43 (0.17)	67.77 (0.17)	67.94 (0.17)	67.93 (0.17)	68.47 (0.17)	0.001
Fat %E	21.50 (0.18)	20.61 (0.18)	20.13 (0.18)	19.88 (0.18)	18.83 (0.18)	< 0.001
Total protein %E	11.06 (0.04)	11.61 (0.04)	11.91 (0.04)	12.18 (0.04)	12.69 (0.04)	< 0.001
Animal protein %E	2.96 (0.04)	3.06 (0.04)	3.15 (0.04)	3.26 (0.04)	3.19 (0.04)	< 0.001
Plant protein %E	8.10 (0.04)	8.54 (0.04)	8.76 (0.04)	8.92 (0.04)	9.49 (0.04)	< 0.001

Values are reported as mean and standard error

P-values derived were derived from analysis of covariance test, after adjustment for age, physical activity (METs min/day) and wealth score index (except Age) in quintiles of total protein intake per body weight (gr/kg)

Additional adjustments:

The significance level was considered P < 0.05

*BMI* body mass index, *HDL-c* high-density lipoprotein *LDL-c* low-density lipoprotein

^*^Adjusted for smoking (Yes/No), monounsaturated fatty acids (MUFAs), polyunsaturated fatty acids (PUFAs) and saturated fatty acids (SFAs) (gr/1000 kcal), sodium and potassium (mg/1000 kcal), diabetes mellitus (Yes/No) and dyslipidemia (Yes/No) and treatment for hypertension (Yes/No)

^†^Adjusted for smoking, dyslipidemia (Yes/No) and blood glucose-lowering drugs (Yes/No)

^‡^Adjusted for smoking, MUFAs, PUFAs, SFAs, diabetes mellitus and treatment for dyslipidemia (Yes/No)

### Association of protein intake with metabolic syndrome and its components

Table 4 shows the odds ratio and 95% confidence interval for metabolic syndrome and its components, according to the groups of daily protein intake per body weight as total protein, animal protein, and plant protein intake in men and women after adjusting for potential covariates. A dose–response association between total protein intake and abdominal obesity, increased blood pressure, reduced HDL-C, elevated serum triglycerides, impaired glucose levels, and metabolic syndrome was observed in both genders. Men in the highest quintile group of total dietary protein intake had a lower prevalence of abdominal obesity (OR: 0.12, 95% CI 0.09–0.15, P-trend < 0.001), reduced prevalence of increased blood pressure (OR: 0.57, 95% CI 0.43–0.74, P-trend < 0.001), decreased prevalence of reduced HDL-C (OR: 0.51, 95% CI 0.40–0.64, P-trend < 0.001), lower prevalence of elevated serum triglycerides (OR: 0.47, 95% CI 0.37–0.59, P-trend < 0.001), reduced prevalence of high glucose levels (OR: 0.52, 95% CI 0.38–0.72, P-trend < 0.001), and decreased prevalence of having metabolic syndrome (OR: 0.24, 95% CI 0.18–0.33, P-trend < 0.001) in comparison with the lowest quintile group. Women in the highest quintile of total protein intake also had a lower prevalence of abdominal obesity (OR: 0.15, 95% CI 0.11–0.20, P-trend < 0.001), decreased prevalence of increased blood pressure (OR: 0.55, 95% CI 0.45–0.68, P-trend < 0.001), lower prevalence of reduced HDL-C (OR: 0.65, 95% CI 0.55–0.78, P-trend < 0.001), reduced prevalence of elevated serum triglycerides (OR: 0.66, 95% CI 0.54–0.81, P-trend < 0.001), lower prevalence of high glucose levels (OR: 0.66, 95% CI 0.53–0.82, P-trend = 0.001), and decreased prevalence of metabolic syndrome (OR: 0.42, 95% CI 0.0.34–0.51, P-trend < 0.001) compared with the lowest quintile group.

**Table 4:** Odds ratios and 95% confidence intervals for metabolic syndrome components according to quintiles of protein intakes per body weight (gr/kg) in men and women

Men						
	Q1	Q2	Q3	Q4	Q5	P for Trend^†^
Total protein						
N = 3659	731	732	732	732	732	
Median intake (gr/kg BW)	0.94	1.23	1.48	1.74	2.17	
Median intake (%E)	11.44	11.77	11.93	12.31	12.77	
Elevated waist circumference	Ref	0.74 (0.59–0.92)^*^	0.47 (0.38–0.59)^*^	0.34 (0.27–0.42)^*^	0.12 (0.09–0.15)^*^	< 0.001
Hypertension	Ref	0.83 (0.65–1.05)	0.78 (0.61–0.99)^*^	0.75 (0.58–0.96)^*^	0.57 (0.43–0.74)^*^	< 0.001
Increased TG	Ref	0.94 (0.76–1.16)	0.73 (0.58–0.91)^*^	0.71 (0.57–0.89)^*^	0.47 (0.37–0.59)^*^	< 0.001
Reduced HDL-c	Ref	1.04 (0.84–1.29)	0.85 (0.68–1.06)	0.76 (0.61–0.95)^*^	0.51 (0.40–0.64)^*^	< 0.001
Elevated blood glucose	Ref	1.02 (0.78–1.33)	0.61 (0.45–0.82)^*^	0.73 (0.55–0.98)^*^	0.52 (0.38–0.72)^*^	< 0.001
Metabolic syndrome	Ref	0.85 (0.67–1.06)	0.61 (0.48–0.77)^*^	0.56 (0.44–0.71)^*^	0.24 (0.18–0.33)^*^	< 0.001
Animal protein						
Median intake (gr/kg BW)	0.19	0.30	0.39	0.51	0.73	
Median intake (%E)	1.83	2.71	3.24	3.82	5.19	
Elevated waist circumference	Ref	0.82 (0.66–1.02)	0.71 (0.56–0.88)^*^	0.65 (0.52–0.81)^*^	0.32 (0.25–0.41)^*^	< 0.001
Hypertension	Ref	0.99 (0.78–1.28)	1.13 (0.88–1.47)	1.02 (0.77–1.33)	0.77 (0.57–1.05)	0.076
Increased TG	Ref	0.92 (0.74–1.15)	0.72 (0.57–0.90)^*^	0.77 (0.61–0.92)^*^	0.72 (0.55–0.93)^*^	0.016
Reduced HDL-c	Ref	0.83 (0.67–1.04)	0.86 (0.69–1.09)	0.89 (0.70–1.14)	0.74 (0.57–0.96)^*^	0.098
Elevated blood glucose	Ref	1.01 (0.76–1.35)	0.97 (0.71–1.31)	0.92 (0.67–1.27)	0.81 (0.57–1.15)	0.192
Metabolic syndrome	Ref	0.86 (0.67–1.09)	0.74 (0.57–0.95)^*^	0.77 (0.59–1.00)	0.50 (0.37–0.68)^*^	< 0.001
Plant protein						
Median intake (gr/kg BW)	0.64	0.84	1.04	1.25	1.61	
Median intake (%E)	7.47	8.05	8.41	8.90	9.67	
Elevated waist circumference	Ref	0.70 (0.56–0.88)^*^	0.62 (0.49–0.77)^*^	0.36 (0.28–0.45)^*^	0.20 (0.16–0.25)^*^	< 0.001
Hypertension	Ref	0.97 (0.76–1.24)	0.82 (0.63–1.06)	0.70 (0.53–0.93)^*^	0.68 (0.50–0.91)^*^	0.004
Increased TG	Ref	0.89 (0.71–1.11)	0.89 (0.71–1.12)	0.73 (0.57–0.92)^*^	0.54 (0.41–0.70)^*^	< 0.001
Reduced HDL-c	Ref	0.89 (0.72–1.11)	0.84 (0.67–1.05)	0.75 (0.59–0.95)^*^	0.47 (0.36–0.61)^*^	< 0.001
Elevated blood glucose	Ref	0.91 (0.69–1.21)	0.77 (0.57–1.04)	0.68 (0.50–0.94)^*^	0.54 (0.38–0.78)^*^	< 0.001
Metabolic syndrome	Ref	0.86 (0.68–1.08)	0.76 (0.60–0.98)^*^	0.59 (0.45–0.77)^*^	0.35 (0.25–0.48)^*^	< 0.001

Women						
	Q1	Q2	Q3	Q4	Q5	P for trend^†^
Total protein						
N = 5260	1052	1053	1052	1051	1052	
Median intake (gr/kg)	0.75	1.04	1.27	1.56	2.01	
Median intake (%E)	11.12	11.61	11.88	12.16	12.63	
Elevated waist circumference	Ref	0.62 (0.45–0.85)^*^	0.41 (0.30–0.55)^*^	0.34 (0.25–0.47)^*^	0.15 (0.11–0.20)^*^	< 0.001
Hypertension	Ref	0.91 (0.75–1.11)	0.80 (0.65–0.97)^*^	0.70 (0.57–0.85)^*^	0.55 (0.45–0.68)^*^	< 0.001
Increased TG	Ref	1.01 (0.84–1.21)	0.78 (0.65–0.95)^*^	0.75 (0.62–0.91)^*^	0.66 (0.54–0.81)^*^	< 0.001
Reduced HDL-c	Ref	0.95 (0.80–1.13)	0.78 (0.65–0.93)^*^	0.64 (0.54–0.77)^*^	0.65 (0.55–0.78)^*^	< 0.001
Elevated blood glucose	Ref	0.72 (0.59–0.89)^*^	0.79 (0.64–0.96)^*^	0.76 (0.62–0.93)^*^	0.66 (0.53–0.82)^*^	0.001
Metabolic syndrome	Ref	0.81 (0.67–0.97)^*^	0.63 (0.52–0.76)^*^	0.56 (0.46–0.68)^*^	0.42 (0.34–0.51)^*^	< 0.001
Animal protein						
Median intake (gr/kg)	0.13	0.22	0.30	0.41	0.59	
Median intake (%E)	1.51	2.31	2.89	3.52	4.57	
Elevated waist circumference	Ref	1.14 (0.87–1.50)	1.18 (0.90–1.54)	0.77 (0.59–0.99)^*^	0.59 (0.46–0.75)^*^	< 0.001
Hypertension	Ref	1.04 (0.86–1.27)	0.78 (0.64–0.96)^*^	0.89 (0.72–1.11)	0.85 (0.68–1.07)	0.097
Increased TG	Ref	1.09 (0.90–1.32)	1.09 (0.89–1.32)	1.21 (0.98–1.48)	1.05 (0.84–1.31)	0.793
Reduced HDL-c	Ref	0.94 (0.78–1.12)	1.09 (0.91–1.30)	0.91 (0.75–1.10)	0.85 (0.70–1.04)	0.075
Elevated blood glucose	Ref	0.97 (0.78–1.20)	1.04 (0.84–1.29)	0.95 (0.76–1.19)	1.01 (0.79–1.28)	0.921
Metabolic syndrome	Ref	0.98 (0.81–1.18)	0.96 (0.79–1.16)	0.90 (0.73–1.10)	0.88 (0.71–1.10)	0.148
Plant protein						
Median intake (gr/kg)	0.53	0.74	0.92	1.14	1.58	
Median intake (%E)	7.72	8.17	8.69	9.09	10.07	
Elevated waist circumference	Ref	0.68 (0.49–0.93)^*^	0.50 (0.37–0.68)^*^	0.41 (0.31–0.56)^*^	0.19 (0.14–0.25)^*^	< 0.001
Hypertension	Ref	0.91 (0.75–1.12)	0.79 (0.64–0.98)^*^	0.74 (0.64–0.98)^*^	0.62 (0.48–0.79)^*^	< 0.001
Increased TG	Ref	0.83 (0.69–1.01)	0.68 (0.55–0.83)^*^	0.63 (0.51–0.78)^*^	0.56 (0.45–0.71)^*^	< 0.001
Reduced HDL-c	Ref	0.92 (0.77–1.10)	0.78 (0.65–0.94)^*^	0.75 (0.62–0.91)^*^	0.63 (0.51–0.77)^*^	< 0.001
Elevated blood glucose	Ref	0.85 (0.69–1.04)	0.72 (0.57–0.89)^*^	0.81 (0.64–1.02)	0.68 (0.53–0.87)^*^	0.006
Metabolic syndrome	Ref	0.82 (0.68–0.99)^*^	0.61 (0.50–0.75)^*^	0.60 (0.48–0.74)^*^	0.41 (0.33–0.52)^*^	< 0.001

All values are reported as (OR and 95% CIs) and were derived from a logistic regression model after adjustment for age, physical activity (METs min/day) and wealth score index

Additional adjustments:

Smoking (Yes/No), monounsaturated fatty acids (MUFAs), polyunsaturated fatty acids (PUFAs) and saturated fatty acids (SFAs) (gr/1000 kcal), sodium and potassium (mg/1000 kcal), diabetes mellitus (Yes/No) and dyslipidemia (Yes/No) for hypertension and metabolic syndrome models

Smoking, MUFAs, PUFAs, SFAs and diabetes mellitus for increased TG and reduced HDL-c models

Smoking and dyslipidemia for elevated blood glucose model

The significance level was considered P < 0.05

*TG* triglycerides, *HDL-c* high-density lipoprotein

^*^P-value < 0.05, derived from a multivariate logistic regression model

^†^P for trend derived from multivariate logistic regression model using the median protein intake per body weight (gr/kg) for each quintile as the continuous variable

After dividing dietary proteins into animal and plant sources, animal protein intake was associated with a lower risk of having abdominal obesity in both genders (P-trend < 0.001). Animal protein intake was also related to a reduced prevalence of having metabolic syndrome (P-trend < 0.001) and lower prevalence of having elevated serum triglycerides (P-trend = 0.016) only in men. Plant protein was significantly related to decreased prevalence metabolic syndrome and all of its components in men and women. Men in the highest quintile group of plant protein intake had a lower prevalence of abdominal obesity (OR: 0.20, 95% CI 0.16–0.25, P < 0.001), reduced prevalence of increased blood pressure (OR: 0.68, 95% CI 0.50–0.91, P = 0.004), decreased prevalence of reduced HDL-C (OR:0.47, 95% CI 0.36–0.61, P < 0.001), reduced prevalence of elevated serum triglycerides (OR: 0.54, 95% CI 0.41–0.70, P < 0.001), lower prevalence of high glucose levels (OR: 0.54, 95% CI 0.38–0.78, P < 0.001), and reduced prevalence of metabolic syndrome (OR: 0.35, 95% CI 0.25–0.48, P < 0.001) than the lowest quintile group. Women in the highest quintile group of plant protein intake had a lower prevalence of abdominal obesity (OR: 0.19, 95% CI 0.14–0.25, P < 0.001), decreased prevalence of increased blood pressure (OR: 0.62, 95% CI 0.48–0.79, P < 0.001), lower prevalence of reduced HDL-C (OR: 0.63, 95% CI 0.51–0.77, P < 0.001), reduced prevalence of elevated serum triglycerides (OR: 0.56, 95% CI 0.45–0.71, P < 0.001), lower prevalence of high glucose levels (OR: 0.68, 95% CI 0.53–0.87, P = 0.006), and decreased risk of having metabolic syndrome (OR: 0.41, 95% CI 0.33–0.52, P < 0.001) than the lowest quintile.

### Association of total dietary protein intake with body composition

Table 5 shows the mean and standard error of lean tissue (kg), adipose tissue (kg), and the percentage of body fat in different parts of the body according to quintiles of total protein intake in men (n = 1758) and women (n = 2419). Higher protein intake was associated with lower body fat percentage (FATP) and fat mass (FM) in the whole body, particularly the trunk, in men and women. Men and women in the highest quintile of total protein intake had 7.4% less (P < 0.001) overall body fat percentage and less fat percentage in the trunk (6.4% and 8.7%, respectively) (P < 0.001) than the lowest quintile group. Also the amount of fat free mass was negatively associated with total protein intake and individuals in the highest quintile of total protein intake had a lower amount (men: 7.1 and women: 4.5 kg) of total free fat mass (P < 0.001).

**Table 5:** Body composition in quintiles of total protein intake per body weight (gr/kg) in men and women (SE)

		Q1	Q2	Q3	Q4	Q5	P-value
Men (n = 1758)							
Right arm	FFM (kg)	3.28 (0.02)	3.19 (0.02)	3.05 (0.02)	2.93 (0.02)	2.75 (0.02)	< 0.001
	FATM (kg)	0.89 (0.01)	0.78 (0.01)	0.68 (0.01)	0.60 (0.01)	0.49 (0.01)	< 0.001
	FATP (%)	20.49 (0.26)	18.95 (0.26)	17.43 (0.26)	16.48 (0.26)	14.60 (0.26)	< 0.001
Left arm	FFM (kg)	3.25 (0.02)	3.19 (0.02)	3.06 (0.02)	2.91 (0.02)	2.74 (0.02)	< 0.001
	FATM (kg)	0.94 (0.01)	0.82 (0.01)	0.70 (0.01)	0.62 (0.01)	0.50 (0.01)	< 0.001
	FATP (%)	21.50 (0.29)	19.60 (0.28)	17.84 (0.28)	16.91 (0.28)	14.82 (0.29)	< 0.001
Right leg	FFM (kg)	9.95 (0.06)	9.74 (0.06)	9.36 (0.06)	9.04 (0.06)	8.62 (0.06)	< 0.001
	FATM (kg)	2.58 (0.04)	2.24 (0.04)	1.98 (0.04)	1.78 (0.04)	1.43 (0.05)	< 0.001
	FATP (%)	19.78 (0.29)	18.08 (0.29)	16.73 (0.28)	15.77 (0.28)	16.69 (0.28)	< 0.001
Left leg	FFM (kg)	9.79 (0.06)	9.53 (0.06)	9.17 (0.06)	8.82 (0.06)	8.38 (0.06)	< 0.001
	FATM (kg)	2.54 (0.04)	2.22 (0.04)	1.94 (0.04)	1.76 (0.04)	1.42 (0.04)	< 0.001
	FATP (%)	19.75 (0.27)	18.19 (0.27)	16.80 (0.27)	15.95 (0.27)	13.95 (0.27)	< 0.001
Trunk	FFM (kg)	31.20 (0.19)	30.88 (0.18)	29.96 (0.18)	28.98 (0.18)	27.74 (0.19)	< 0.001
	FATM (kg)	11.41 (0.21)	10.02 (0.21)	8.63 (0.21)	7.75 (0.21)	6.23 (0.21)	< 0.001
	FATP (%)	25.78 (0.40)	23.55 (0.40)	21.29 (0.40)	20.18 (0.40)	19.37 (0.40)	< 0.001
Total body	FFM (kg)	57.47 (0.35)	56.53 (0.35)	54.63 (0.35)	52.70 (0.35)	50.28 (0.35)	< 0.001
	FATM (kg)	18.35 (0.34)	16.05 (0.34)	13.87 (0.34)	12.46 (0.34)	10.04 (0.34)	< 0.001
	FATP (%)	23.28 (0.34)	21.29 (0.34)	19.36 (0.34)	18.33 (0.34)	15.84 (0.34)	< 0.001
Women (n = 2419)							
Right arm	FFM (kg)	2.25 (0.01)	2.20 (0.01)	2.13 (0.01)	2.07 (0.01)	1.99 (0.01)	< 0.001
	FATM (kg)	1.57 (0.02)	1.39 (0.02)	1.25 (0.02)	1.09 (0.02)	0.89 (0.02)	< 0.001
	FATP (%)	39.40 (0.36)	37.27 (0.36)	35.46 (0.36)	33.17 (0.36)	29.45 (0.36)	< 0.001
Left arm	FFM (kg)	2.31 (0.01)	2.24 (0.01)	2.16 (0.01)	2.09 (0.01)	1.99 (0.01)	< 0.001
	FATM (kg)	1.67 (0.02)	1.47 (0.02)	1.32 (0.02)	1.14 (0.02)	0.93 (0.02)	< 0.001
	FATP (%)	40.05 (0.36)	37.95 (0.36)	36.17 (0.36)	33.83 (0.36)	30.15 (0.36)	< 0.001
Right leg	FFM (kg)	7.50 (0.03)	7.28 (0.03)	7.09 (0.03)	6.86 (0.03)	6.60 (0.03)	< 0.001
	FATM (kg)	5.69 (0.05)	5.29 (0.05)	4.94 (0.05)	4.55 (0.05)	4.06 (0.05)	< 0.001
	FATP (%)	42.47 (0.21)	41.53 (0.21)	40.49 (0.21)	39.38 (0.21)	37.51 (0.21)	< 0.001
Left leg	FFM (kg)	7.42 (0.03)	7.19 (0.03)	7.00 (0.03)	6.77 (0.03)	6.50 (0.03)	< 0.001
	FATM (kg)	5.61 (0.05)	5.23 (0.05)	4.89 (0.05)	4.50 (0.05)	4.02 (0.05)	< 0.001
	FATP (%)	42.41 (0.20)	41.59 (0.20)	40.54 (0.20)	39.40 (0.20)	37.61 (0.20)	< 0.001
Trunk	FFM (kg)	25.19 (0.10)	24.78 (0.10)	24.22 (0.10)	23.69 (0.10)	23.05 (0.10)	< 0.001
	FATM (kg)	13.18 (0.18)	11.87 (0.18)	10.98 (0.18)	9.71 (0.18)	8.03 (0.18)	< 0.001
	FATP (%)	33.29 (0.34)	31.49 (0.33)	30.21 (0.33)	28.07 (0.33)	24.68 (0.34)	< 0.001
Total body	FFM (kg)	44.67 (0.19)	43.69 (0.19)	42.69 (0.19)	41.47 (0.19)	40.14 (0.19)	< 0.001
	FATM (kg)	27.71 (0.34)	25.24 (0.34)	23.36 (0.33)	20.96 (0.33)	17.90 (0.34)	< 0.001
	FATP (%)	37.31 (0.28)	35.78 (0.28)	34.54 (0.28)	32.74 (0.28)	29.90 (0.28)	< 0.001

All values are presented as mean and standard error

P-values were obtained using analysis of covariance test in quintiles of total protein intake (g/kg) after adjusting for age, physical activity, and wealth index

The significance level was considered P < 0.05

*FFM* free fat mass, *FATM* fat mass, *FATP* fat percentage

## Discussion

Metabolic syndrome is one of the major public health problems in the last century, which is due to the changes in lifestyle and dietary and behavioral patterns. In the present study, we assessed the relationship of the amount of daily protein intake per body, as three types of total, animal and plant protein, with the risk of having metabolic syndrome and its components. The result of this study showed a dose–response pattern and a higher consumption of total protein seemed to be associated with a lower prevalence of metabolic syndrome in both genders. Plant protein intake was negatively related with the prevalence of having metabolic syndrome and all of the components in men and women. In addition, higher animal protein intake was related to a lower prevalence of abdominal obesity, a declined risk of having increased TG, and a decreased prevalence of metabolic syndrome in men, as well as a reduced prevalence of abdominal obesity in women.

### Protein intake and metabolic syndrome

The present study showed that men and women in the higher quintiles of total protein intake had lower odds of having metabolic syndrome (Men: Q2: 15%, Q3: 39%, Q4: 44%, Q5: 76%; Women: Q2: 19%, Q3: 37%, Q4: 44%, Q5: 58%) compared to those in the lowest quintile of total protein intake. Also higher plant protein intake was associated with a lower incidence of metabolic syndrome. Furthermore, higher animal protein intake was related to a 50% reduction in the prevalence of metabolic syndrome in men. One study of people over the age of 51 years in Korea found that consuming less than 0.8 g of protein per kg body weight per day was associated with a higher risk of metabolic disorders in adults. They suggested that consuming 1.2 g of protein per kg body weight per day or more could reduce metabolic risks [35]. Pasiakos et al. suggested that daily protein intake higher than the recommended dietary value (RDA) was safe and appeared to be a valid value for improving cardiometabolic health [36]. Another study found that a plant-rich diet and an animal-rich diet, in the form of an isocaloric diet, had a similar effect on improving inflammatory cardiovascular variables, meaning that a plant-rich diet was not superior to an animal-rich diet [37].On the other hand, Shang et al. stated that consuming higher total protein and animal protein was associated with an increased risk of metabolic syndrome, and only plant protein was associated with lower risks [38]. Another study found that the high dietary protein to carbohydrates ratio was associated with an increased incidence of metabolic syndrome only in men but no association was found in women [39].

### Protein intake and hypertension

Based on our result, mean systolic and diastolic blood pressure seem to be lower in men and women who consumed more total protein per body weight. However, after adjusting for more variables, the changes were observed only in men. In addition, higher total protein intake in both genders was associated with a reduced prevalence (Men: Q2: 17%, Q3: 22%, Q4: 25%, Q5: 43%; Women: Q2: 9%, Q3: 20%, Q4: 30%, Q5: 45%) of hypertension. After classifying the protein source into animal and plant, the reduced prevalence of hypertienson was observed only for plant proteins. A study by Mehrbani et al. showed that regular consumption of protein, especially plant protein, was related to lower mean systolic and diastolic blood pressure [40]. A study of adults in the Netherlands also found that total dietary protein and animal protein intake was not related to blood pressure, and only plant protein was associated with lower blood pressure [41]. On the other hand, some studies have linked animal protein to blood pressure. Results of a study in Japan showed that total protein intake was inversely related to diastolic blood pressure levels. They also reported a significant inverse relationship between animal protein intake and systolic blood pressure levels and the risk of cardiovascular disease [42].

### Protein intake and impaired blood glucose

The current study showed that men who received more total protein per day per body weight had lower mean fasting glucose levels in a dose–response pattern, but after adjusting for more variables, the differences were no longer significant. However, it was observed that total protein intake was inversely associated with the prevalence of impaired glucose levels in both men and women. Men and women in higher quintiles of total protein intake had lower odds of having elevated blood glucose levels (Men: Q3: 39%, Q4: 27%, Q5: 48%; Women: Q2: 28%, Q3: 21%, Q4: 24%, Q5: 34%) compared to those in the lowest quintile of total protein intake. Although the plant protein appeared to be related to a lower chance of having impaired glucose levels, no association was observed for animal protein. Recently, studies have been conducted on the role of protein intake and the risk of type 2 diabetes, which showed the favorable effect of a high protein diet on improving glucose homeostasis in short-term experiments. According to a meta-analysis, total protein and animal protein intake were risk factors for type 2 diabetes in both genders, while plant protein had a protective role in females [43]. A study in Korea found that the risk of impaired fasting glucose levels was significantly higher when the daily protein intake was less than 0.8 g per body weight compared to the higher daily protein intake [35]. In contrast, the study of Sluijs et al. showed that high intake of total protein and animal protein, but not plant protein, was related to a higher risk of diabetes [44].

### Protein intake and lipids profile

This study showed that total protein intake in both genders was negatively related to the serum triglyceride level and positively associated with serum HDL-c level. As well, it was observed that higher total protein intake was associated with a decrease in the prevalence of elevated triglyceride levels in both men and women. After sorting the dietary protein intake based on sources, animal protein was associated with decreased risk of having elevated triglyceride levels only in men, but plant protein was related to a lower risk of having increased triglyceride levels in both genders. In addition, total protein intake was negatively associated with the chance of having decreased HDL-c levels in both genders. Animal protein intake did not have any significant association with the prevalence of decreased HDL-c in any gender, but a higher plant protein intake was related to a decreased risk of having decreased HDL-c in both genders. One study found that daily protein intake higher than the recommended dietary intake (RDA), between 1 and 1.5 g per kg body weight, was associated with increased high-density lipoprotein levels [36]. Another meta-analysis stated that plant protein intake, in comparison to animal protein intake, was associated with improved lipid profile including increased HDL-c, and decreased triglycerides in people with hypercholesterolemia [45]. A study by Oh et al. showed that daily protein intake lower than 0.8 g per body weight, in comparison to higher intakes, increased the risk of having impaired serum triglyceride levels [35].

### Protein intake and obesity

It was observed in our results that men and women with higher total protein intake per body weight had lower weight, body mass index, waist circumference, hip circumference, and waist-to-hip ratio in a significant manner. Moreover, it was observed that a higher intake of total dietary protein was associated with a dose–response pattern in reducing the prevalence of having abdominal obesity. After dividing proteins based on their source, both plant and animal protein intake appeared to be associated with a reduced prevalence of abdominal obesity in both genders. Previous studies showed that body mass index and waist circumference were negatively related to protein intake, and central obesity was lower in subjects on a high-protein diet [36]. Park et al. also reported an inverse relationship of total protein, plant and animal protein intake with body mass index and waist circumference in individuals over the age of 60 years [46]. However, some studies have reported different results regarding the association of total protein and animal protein intake with obesity [25, 47]. Shang et al. stated that a higher intake of plant protein was associated with lower waist circumference and body weight, but increased intake of total protein and animal protein was related to higher waist circumference [38].

In addition, based on the results of this study, it seems that individuals of both genders who consumed higher total protein had lower fat mass and lower fat percentage in all areas of their body, especially in the trunk area. The study of Green et al. also found that higher protein intake, even in the absence of calorie restriction, was associated with lower total body fat percentage and lower body fat percentage [48]. Moreover, the study of Soenon et al. showed that increased daily protein intake was negatively associated with body fat percentage, and positively related to free-fat mass during 3 months of intervention without energy restriction or increased physical activity [49]. By contrast, in research by Vinknes et al. a weak positive relationship between the energy percentage intake from dietary protein and the body fat percentage was observed [50].

### Protein intake and possible mechanisms

To date, various mechanisms have been proposed for the role of protein intake in reducing the risk of developing metabolic syndrome and its components, as well as the differences in the effect of plant and animal protein intake. Firstly, the protein content of the diet can cause various changes in the digestive process of the meals, such as increasing satiety and delaying gastrointestinal emptying, thereby results in a decrease in food intake and an increase in postprandial heat production. Therefore, it can decrease obesity and improve insulin resistance, glucose homeostasis, and lipids profile [45]. Not only the amount of consumed protein affects the metabolic profile, but also the source and quality of dietary protein can make difference. Plant source of protein includes Fruits, vegetables, nuts, legumes and whole grains, which contain fiber, vitamins, minerals, antioxidants, phenolic compounds, and unsaturated fatty acids. Many clinical trials and studies have demonstrated the effect of these components on improving insulin sensitivity and blood pressure [51]. For instance, a study by Motamedi et al. on the Fasa cohort data showed that the Mediterranean dietary score and a healthy eating index-2015 was associated with a lower risk of having hypertension. They also indicated that higher adherence to the Mediterranean dietary score and healthy eating index-2015 was associated with a decrease in the obesity-related parameters, including weight, Waist circumference, BMI, and hip circumference [52]. Furthermore, isoflavones in plant-based diets, which regulate the expression of sterol regulatory element-binding protein (SREBP)-2, improve cholesterol clearance from serum [45]. Also fiber has a role in reducing total cholesterol by affecting LDL-c through various mechanisms. The study of Momenizadeh et al. showed that the consumption of oat bread as a source of Beta-glucan, had hypocholesterolemic effects and was able to reduce cholesterol levels and BMI [53].

Other potential explanations for the differences between the sources of protein can be attributed to the amino acids composition of the proteins. For example, the role of glycine, tryptophan, and tyrosine, which are predominant in animal protein sources, have been proved in modulating vascular tone and endothelial function and they are associated with lower blood pressure. Glutamic acid, which is greatly in plants, is related to lower atrial stiffness [25]. Another difference between animal and plant protein can be related to antinutritional factors including glucosinolates, trypsin inhibitors, hemagglutinins, tannins, phytates, and gossypol, which may form by heating or alkaline processing of animal and vegetable protein foods because they can change the digestibility and bioavailability of proteins [54].

### Protein content of processed foods and metabolic syndrome

According to NOVA food classification system, foods based on the nature, extent, and purpose of industrial processing have been categorized in four groups: (i) Unprocessed or minimally processed foods (MPFs); (ii) Processed culinary ingredients; (iii) Processed foods; (iv) Ultra-processed foods (UPfs) [12]. A study by Matos et al. has reported a comprehensive list of NOVA food classification. Fresh/frozen fruits and vegetables, fresh meat, fresh milk, eggs, nuts, and whole-grain are some examples of MPFs; and UPFs can be addressed as industrial formulas with multiple ingredients, including sweetened beverages, refined cereal and bread, processed cheese and processed meats products, pizza, and confectionary [55]. Previous studies have shown that dietary patterns rich in UPFs are associated with increased risk of several cardiometabolic disorders such as metabolic syndrome, cardiovascular diseases, diabetes, and obesity [56], while the strong inverse relationship of MPFs intake and obesity has been observed [57]. UPFs are characterized by low nutritional quality, high energy density, which included a high amount of simple carbohydrates, saturated fats, and sodium; while they contain a lower amount of fiber, vitamins, minerals, and proteins in comparison with MPFs [56]. Salome et al. reported that higher energy intake from MPFs was related to higher animal protein intake, better plant protein diversity, increased diet quality, and remarkably decreased risk of cardiometabolic diseases. It could be expected since the major source of animal-based proteins, such as poultry, fish, red meat, yogurt, and milk are categorized as MPFs. Whereas, in higher UPFs consumers, intake of plant-based protein was higher because of higher intake of bread and refined grains. Higher consumption of UPFs was related to lower protein intakes as increased intake of miscellaneous foods groups low in protein like sweetened drinks, pastries, or confectionary [58]. Pervious studies have reported that diluation of protein in diet by either fat or carbohydrate will result in excessive food intake to reach the protein intake target, as a known phonomenon named ‘protein leverage’. An increase in the consuption of protein-dulated foods, such as ultra-processed enegy dense foods, is suggested as a driver for increasing obesity in China an the US [59]. In addition, is it reported that habitually consuming high protein densty diet was associated with higher diet quality and dietary micronutrient in healthy young adults [60]. Therefore, it seems that the protein content of the diet is in a close relationship with diet quality, and high protein diets usually consist of less processed foods. It could be an explanation for the effect of high protein diets on preventing or controlling metabolic syndrome. Evaluating diet quality regarding plant- or animal-based protein may be a missing key in studying the role of protein sources in metabolic syndrome. Further studies are suggested to experiment with the combined impact of the protein source and diet quality in metabolic syndrome to illuminate the unknown sides such as their priority in diet planning and distinguishing their role as a single factor in preventing metabolic syndrome.

### Limitations and strengths of this study

To our knowledge, this study is the first one to analyze the association of protein intake from food sources with metabolic syndrome risk factors using a large sample of Iranian adults including 8922 subjects (3660 men and 5262 women), with a wide range of people considering socio-economic indices. Another positive point of this study is a face-to-face interview for data collection of FFQ and conducting the anthropometric measurements instead of self-reporting, which increases the reliability of data. However, this study also had some limitations which can be pointed out to be cross-sectional, which may not well indicate the effect of protein intake on reducing the risk of developing metabolic syndrome and its components in the long term. Another possible limitation was the use of a food frequency questionnaire (FFQ) to collect nutritional information, which could be susceptible to recall bias.

## Conclusion

According to the results of this study, a higher intake of total protein seemed to be associated with a lower prevalence of metabolic syndrome and all of its components in both genders. After dividing dietary proteins based on their source to plant and animal protein, men and women in higher quintiles of plant protein intake had a reduced prevalence of metabolic syndrome and its components. In addition, animal protein in the results of the current study was associated with a reduced prevalence of metabolic syndrome and decreased risk of having elevated TG in men, and a lower prevalence of abdominal obesity in both genders. However, due to conflicting results of other studies, animal protein must be consumed with caution, and further studies are needed to discover the association of animal protein intake and metabolic syndrome.

## Data Availability

The datasets used and/or analyzed during the current study are available from the corresponding author on request.
